# Economic burden of malaria and predictors of cost variability to rural households in south-central Ethiopia

**DOI:** 10.1371/journal.pone.0185315

**Published:** 2017-10-11

**Authors:** Alemayehu Hailu, Bernt Lindtjørn, Wakgari Deressa, Taye Gari, Eskindir Loha, Bjarne Robberstad

**Affiliations:** 1 Centre for International Health, Department of Global Public Health and Primary Care, University of Bergen, Bergen, Norway; 2 Department of Reproductive Health and Health Service Management, School of Public Health, Addis Ababa University, Addis Ababa, Ethiopia; 3 Department of Preventive Medicine, School of Public Health, Addis Ababa University, Addis Ababa, Ethiopia; 4 School of Public and Environmental Health, Hawassa University, Hawassa, Ethiopia; 5 Center for Intervention Science in Maternal and Child Health (CISMAC), University of Bergen, Bergen, Norway; Centro de Pesquisas Rene Rachou, BRAZIL

## Abstract

**Background:**

While recognizing the recent remarkable achievement in the global malaria reduction, the disease remains a challenge to the malaria endemic countries in Africa. Beyond the huge health consequence of malaria, policymakers need to be informed about the economic burden of the disease to the households. However, evidence on the economic burden of malaria in Ethiopia is scanty. The aims of this study were to estimate the economic burden of malaria episode and to identify predictors of cost variability to the rural households.

**Methods:**

A prospective costing approach from a household perspective was employed. A total of 190 malaria patients were enrolled to the study from three health centers and nine health posts in Adami Tullu district in south-central Ethiopia, in 2015. Primary data were collected on expenditures due to malaria, forgone working days because of illness, socioeconomic and demographic situation, and households’ assets. Quantile regression was applied to predict factors associated with the cost variation. Socioeconomic related inequality was measured using concentration index and concentration curve.

**Results:**

The median cost of malaria per episode to the household was USD 5.06 (IQR: 2.98–8.10). The direct cost accounted for 39%, while the indirect counterpart accounted for 61%. The history of malaria in the last six months and the level of the facility visited in the health system predominantly influenced the direct cost. The indirect cost was mainly influenced by the availability of antimalarial drugs in the health facility. The concentration curve and the concentration index for direct cost indicate significant pro-rich inequality. *Plasmodium falciparum* is significantly more costly for households compared to *Plasmodium vivax*.

**Conclusion:**

The economic burden of malaria to the rural households in Ethiopia was substantial—mainly to the poor—indicating that reducing malaria burden could contribute to the poverty reduction as well.

## Introduction

An intensified and increased commitment and financial allocation for malaria prevention and control measures have reduced the burden of malaria mortality rate among under five children by 29% globally within five years, since 2010 [1]. Despite being a largely preventable and treatable disease, malaria accounts for about 212 million of cases and 429,000 deaths globally in 2015 alone [1]. Sub-Saharan Africa continues to bear a disproportionate share of the global burden with more than 90% of malaria cases and deaths [2] with Ethiopia as one of the hardest-hit countries. According to the 2016 World Malaria Report, more than 1.8 million of microscopically confirmed cases were reported in the country [1].

Beyond the huge health consequence, malaria imposes a heavy economic burden on individuals, households and the entire economy [3]. Malaria alone reduces the potential economic growth rate by 1.3% per year in some African countries as a single disease [4]. Gallup and Sachs claimed that, at macro-level, malaria and poverty are intimately connected, in which the malaria is the main contributor to poverty [4], while at micro or household level, the causal link yet remains unclear.

Unlike most of the other African malaria endemic countries, malaria follows a unique epidemiological pattern in Ethiopia. For example, the parasite transmission is seasonal at low to moderate intensity, the national prevalence is estimated to be less than 0.5% [5], and the contribution of *Plasmodium vivax* is substantially high: about 40% of the cases. These factors contribute to a uniquely unstable nature of the transmission pattern in the country, and all age groups of the population are therefore susceptible to severe malaria. These consequently not only make the malaria prevention and control program in Ethiopia more challenging, but it also makes the economic impact to the household potentially overwhelming [3, 6–8]. The recurrent and severe form of *Plasmodium falciparum*, and relapsing and pernicious form of *Plasmodium vivax*, expose poor households to further economic impoverishment in the course of getting treatment and repressed productivity [9].

Evidence on the economic burden of malaria is important for prioritization of prevention and treatment service at the national and sub-national levels and facilitates better resource allocation in the health care system [10–12]. However, only a few of these estimates are available and little research has been conducted on the economic burden of malaria on the rural households in Ethiopia. One of the few, a community-based cross-sectional study done by Deressa et al. in Adami Tullu [13], estimated that the mean direct cost of malaria per patient was 1.6 and the indirect counterpart was 4.1 in 2003 United States Dollar (USD). Another study from Tigray, Tembien, by Cropper et al. indicates a total cost ranging from 7 to 24 for adult patients, 7 to 23 for teenage patients, and 4 to12 for children in 1997 USD [6]. This study also indicates that households in such malarious areas are willing to spend about 15% of their annual household income to prevent malaria [6]. Thus, according to these studies, malaria is clearly one of a major cause of economic burden to rural households in Ethiopia.

The Sustainable Development Goals (SDG) propose to reduce malaria cases and death rate by at least 90% and to eliminate malaria in 35 countries by the year 2030 [14]. Ethiopia is one of the countries targeted for the elimination plan. The strategy encompasses three major pillars. One of the pillars is to ensure universal access to malaria prevention, diagnosis, and treatment [14]. However, in order to achieve these targets, the country-level malaria prevention and control program need to be precisely designed towards alleviating the demand side barriers, mainly cost to the household, by way of providing financial risk protection to households during the time of illness [15–17].

From a practical point of view, the inherent trade-offs between health service cost, health service utilization, productivity loss, and socioeconomic status invites debates on user-fees and out-of-pocket expenditures at the point of treatment [18]. On one hand, there has been a tendency to increase user-fees for basic health services as a means to ensure the sustainability of government supported health systems in low-income countries [19]. On the other hand, increasing costs of basic health services may result in deferral or shift from formal health care, mainly amongst the poor [20, 21].

In the last decade, health care payment and financing mechanism in Ethiopia has been through series of reforms, and in particular, for malaria diagnosis and treatment; but, financing still remains irregular across regions [22, 23]. Moreover, evidence regarding the overall economic burden of malaria to the households is scant. The present study estimates the extent of the direct and indirect cost of malaria; and identifies predictors of cost variability to rural households among cases presented in primary health care units in south-central Ethiopia.

## Methodology

### Study setting and participant selection

This costing study was conducted alongside a large cluster randomized controlled trial, which aims to evaluate the effectiveness, cost and cost-effectiveness of the combined use of long-lasting insecticidal nets (LLINs) and indoor residual spraying (IRS) against each intervention alone in preventing malarial infection [24]. The study was conducted in Adami Tullu district in Oromia region of south-central Ethiopia. This area is predominantly agricultural, where households mainly depended on subsistent farming and livestock production for subsistence.

For the costing study, we collected data from villages which were not included in the main trial in order to avoid alteration of the ‘real’ economic burden due to interventions related with the research undertaking [25]. Three rural health centers and nine health posts (i.e three health posts attached to each health centers) were included. From January—December 2015, 190 malaria cases identified in the selected health facilities were included into the study (about 36 cases from each health center and 10 cases from each health post). The health posts are the lowest level in the Ethiopian health care delivery system, and each serve populations of about 5,000, whereas health centers are the next higher level and intended to serve for about 25,000 populations.

### Data collection

A structured closed-ended and partially open-ended pre-tested questionnaire was used. We adopted a household costing tool first prepared by Hansen and Yeung [26]. The questionnaire was prepared in English and then translated to *Afan Oromo* and then back translated to English to check for consistency. The questionnaire had three main sections: general socio-demographic characteristics, direct and indirect cost information, and socioeconomic characteristics.

Data were collected by trained nurses who administered face-to-face interviews to either the head of household or directly to the household member who had the malaria attack. In order to give adequate time for incidents of expenditures related with the malaria episode, the interview was conducted on the 10^th^ day after the patient was examined and treated at the health facility. All cases were confirmed malaria positive (*P*. *falciparum or P*. *vivax*) by either Rapid Diagnostic Test (RDT) or microscopic blood film examination. Mixed cases were excluded from this study.

### Cost of illness estimation

The cost of illness was estimated by identifying, measuring, and valuing the opportunity cost of the forgone resources caused by the malaria. We employed an incidence-based prospective approach by measuring the cost per episode of malaria to the patient and to the household. The cost estimation was done amongst new cases arising in a predefined period. This provides an estimate of the saving that potentially could accrue if the preventive measure is implemented. [25, 27, 28].

#### Measurement

We followed an ingredient based bottom-up approach to identify and measure all costs at patient level. Direct costs measured in this study were all out-of-pocket expenditures on the course of seeking and obtaining malaria treatment by patients. The direct costs were identified and measured in two groups: (1) direct medical costs (diagnosis, medical supplies, malaria drugs, other drugs, and consultation), and (2) direct non-medical costs (food on the way to the treatment facility, transportation, other non-medical supplies and services). All direct cost information was collected in Ethiopian Birr (ETB). Indirect costs were measured in terms of number of forgone working days of the patients due to the malarial illness. Indirect costs due to caregiving for an ill child or any other patients from family members were not included in this study.

#### Valuation

Direct cost was the sum of direct medical costs and direct non-medical costs, and at the outset estimated in monetary values. Indirect cost was valued using a *human capital approach* [25]. Thus, the value of a labor day (the wage rate) was used to convert the workdays lost into monetary value. For adults older than 18 years, the average daily wage rate for agricultural workers was used [29]. According to the 2013 National Labor Force Survey (NLFS) report, the average monthly wage rate for agricultural worker in Ethiopia was ETB 697, which we divided by 20 in order to obtain the daily wage rate of ETB 35. Proportionally, we assume that a teenager's (aged 13 to17 years-old) daily agricultural productivity is half of an adult’s and for children’s (aged 7 to 12 years-old) daily productivity is a quarter of an adult’s. For children less than 7 years-old, we considered the wage rate as negligible and the indirect cost was not estimated. We adopt this framework from a similar labor valuation study in Ethiopia [30].

All costs were converted to USD using the official National Bank of Ethiopia average exchange rate for 2015 (US$1 = ETB 20.5). We used a consumer price index in order to account for annual inflation. The reference year for all cost estimates in this study is 2015 USD [31].

### Statistical analysis

Patient level data analysis were performed using STATA statistical software, version 14 [32]. Average costs information were stratified and presented by the level of health facility (health post and health center). For all cost information, we report the mean with standard deviation (SD), standard error of the mean (SEM), and median with interquartile range (IQR). The data had been examined for the following statistical assumptions: normality, multicollinearity, and heteroscedasticity. To deal with skewed cost data, Kruskall-Wallis and Mann-Whitney test (non-parametric tests) were used to compare the median costs across different socioeconomic quantiles and malaria species (*P*. *vivax* and *P*. *falciparum*). Then, separate quantile regression models were fitted to identify factors associated with variability of median direct and indirect cost of malaria. We performed bootstrapping with 1000 repetitions to estimate 95% confidence intervals for the median cost and robust standard error of the regression coefficients.

Principal components analysis (PCA) was used to construct a wealth index based on household characteristics, such as availability of various household assets, housing conditions, water source, and type of latrine facility [33]. We used the first principal component with an Eigen value of 3.2 in order to rank the household by wealth status. The overall Kaiser-Meyer-Olkin (KMO) measure of sample adequacy was 0.68.

The concentration index was estimated to explore the inequality in mean and median costs of malaria across different socioeconomic status and concentration curves was illustrated to visually present the distribution [34].

### Ethical consideration

All study participants were informed about the objectives of the study and written informed consent was obtained from each participant before interview. Participation in the study was voluntary. The study was approved by the Institutional Review Board (IRB) of the College of Health Sciences at Addis Ababa University, the Ministry of Science and Technology in Ethiopia (ref: 3.10/446/06) and the Regional Committee for Medical and Health Research Ethics, Western Norway (ref: 2013/986/REK Vest). A permit to conduct this study was obtained from Oromia Regional State Health Bureau.

## Results

### Characteristics of the study participants

Table 1 shows a summary of the study household’s characteristics and description of the malaria episodes. Out of the 190 participants responded, 108 (56.8%) of the participants were identifies at the health centers and 82 (43.2%) were identified at the health posts. The mean household size was 5.1 (range from 1 to 14). The majority of the study participants were Oromo (187, 98%), Muslim (169, 89%), farmers (171, 90%), and from male-headed households (187, 98.4%). More than half (110, 57.9%) of the households’ heads had no formal education but they were able to “read and write”, but only 28 (14.7%) had “attended formal education”. The mean age of the malaria patients was 16 year. The mean duration of fever before seeking health care was 1.3 days, and the duration of the malaria episodes was 3.2 days on average.

**Table 1:** Socio-demographic characteristics and the situation of the malaria illness, Adami Tullu district south-central Ethiopia, 2015.

Characteristics	Mean (SD)	Median (IQR)
Age of household head (year)	35.0 (9.2)	35 (28, 40)
Age of the malaria sick member (year)	16.0 (11.8)	14 (6, 22)
Duration of illness (days)	3.2 (0.9)	3 (3, 4)
Duration of fever before seeking health care (days)	1.3 (1.1)	1 (1, 2)
	**n (%)**	
Days between onset of fever and treatment initiation		
Same day	43 (22.6)	
Next day	97 (51.0)	
After two days and more	50 (26.3)	
Severity of the fever (as reported by the patient)		
Mild	31 (16.3)	
Moderate	141 (74.2)	
Severe	18 (9.5)	
Sex of head of the household		
Male	187 (98.4)	
Female	3 (1.6)	
Educational status of head of the household		
Illiterate (Can’t read and write)	52 (27.4)	
Only can read and write	110 (57.9)	
Formal education attended	28 (14.7)	
Occupation of head of the household		
Farmer	171 (90)	
Other economic activity	19 (10)	
Ethnicity of head of the household		
Oromo	187 (98.4)	
Amhara	3 (1.6)	
Religion of head of the household		
Muslim	169 (88.9)	
Orthodox Christian	15 (7.9)	
Protestant Christian	5 (2.6)	
Wakefeta	1 (0.5)	

### Economic burden of malaria: Direct, indirect and total cost

Table 2 shows the summary of the direct, indirect and total cost of malaria amongst those treated at the health center (a), health post (b), and overall for both levels of care (c). The overall total median cost of malaria per episode to the household was USD 5.06 (Bootstrap 95% CI: 4.42–5.69) and mean total cost of USD 6.1 (Bootstrap 95% CI: 5.34–6.86). The direct cost of USD 2.39 (95% CI: 2.58–2.95) accounted for 39% and the indirect cost of USD 3.76 (Bootstrap 95% CI: 1.51–2.99) accounted for 61% of the total cost. Direct medical cost (median = USD 1.56) was 62% higher than the non-medical (median = USD 0.59) counterpart.

**Table 2:** Direct, indirect and total malaria costs to the household (2015 USD) at health centers, health posts, and overall for both level of care, Adami Tullu district south-central Ethiopia.

Cost categories	Median	[IQR: p25	p75]	Mean	SD	SEM
a. Cost at Health Center						
Direct Medical cost	0.83	0.59	1.10	0.93	0.44	0.0426
Malaria testing cost	0.24	0.20	0.24	0.25	0.21	0.0200
Malarial drug cost	0.00	0.00	0.10	0.10	0.20	0.0194
Other drug cost	0.37	0.00	0.51	0.40	0.42	0.0403
Consultation fees	0.15	0.15	0.24	0.18	0.06	0.0060
Direct non-medical cost	1.88	1.15	2.80	1.97	1.22	0.1171
Transportation cost	0.59	0.00	1.24	0.74	0.68	0.0658
Food	0.98	0.39	1.56	1.03	0.75	0.0722
Other Items	0.00	0.00	0.29	0.20	0.29	0.0283
Direct Cost	2.98	2.10	3.80	2.90	1.29	0.1239
Indirect Cost	2.05	0.00	5.25	3.77	5.27	0.5887
Total Cost	4.76	3.20	9.69	6.67	5.12	0.5726
b. Cost at Health Post						
Direct Medical cost	0.00	0.00	0.39	0.30	0.48	0.0525
Malaria testing cost	0.00	0.00	0.00	0.03	0.11	0.0118
Malarial drug cost	0.00	0.00	0.00	0.05	0.14	0.0160
Other drug cost	0.00	0.00	0.39	0.19	0.29	0.0322
Consultation fees	0.00	0.00	0.00	0.03	0.07	0.0079
Direct non-medical cost	1.10	0.00	2.68	1.39	1.34	0.1478
Transportation cost	0.00	0.00	1.46	0.68	0.83	0.0921
Food	0.49	0.00	1.22	0.63	0.66	0.0732
Other Items	0.00	0.00	0.00	0.08	0.25	0.0273
Direct Cost	1.22	0.00	3.22	1.69	1.67	0.1841
Indirect Cost	3.30	1.05	6.28	3.74	3.52	0.4471
Total Cost	5.08	2.66	7.50	5.43	3.77	0.4791
c. Overall cost						
Direct Medical cost	0.59	0.24	0.88	0.67	0.55	0.0401
Malaria testing cost	0.15	0.00	0.24	0.16	0.20	0.0147
Malarial drug cost	0.00	0.00	0.00	0.08	0.18	0.0131
Other drug cost	0.10	0.00	0.39	0.31	0.38	0.0277
Consultation fees	0.15	0.00	0.24	0.12	0.10	0.0073
Direct no-medical cost	1.56	0.49	2.78	1.72	1.30	0.0943
Transportation cost	0.49	0.00	1.46	0.71	0.75	0.0545
Food	0.88	0.00	1.46	0.86	0.74	0.0536
Other Items	0.00	0.00	0.24	0.15	0.28	0.0204
Direct Cost	2.59	0.88	3.51	2.39	1.58	0.1145
Indirect Cost	2.25	0.00	5.80	3.76	4.57	0.3836
Total Cost	5.06	2.98	8.10	6.15	4.61	0.3869

Mean and median cost includes households reporting no expenditure (0)

#### Cost of diagnosis

The overall median diagnostic testing cost was USD 0.15. However, at health post level, the large majority of the patients were tested with RDT and no user-fee was incurred for the diagnosis testing. For example, 73 out of 82 cases had not paid anything for testing. On the other side, at the health center level, 82% of the cases were diagnosed with blood film microscopic examination while the remaining 18% were diagnosed with RDT. The median diagnostic cost at health center level was USD 0.24 (Table 2), and ranged from USD 0.15 to USD 0.49.

#### Cost of antimalarial drug

One hundred fifty-eight (83%) of the patients received the anti-malaria drug directly from the public facility where they were examined and tested, while the others only received the prescription and went back without the antimalarial drug at the public primary health care facility. Out of those examined at the health post level, 11 (12%) cases report that they paid for the antimalarial drug with payment ranging from USD 0.09 to USD 0.58 USD. Of those seen at the health centers, 32 (30%) reported that they paid from USD 0.1 to USD 0.78 for the antimalarial drug. which ranges from USD 0.1 to USD 0.78 (Table 2).

### Predictors of malaria cost variability

Table 3 presents the multiple quantile regression coefficients with 95% confidence interval for different factors associated with variability in costs of malaria. The household’s socioeconomic status (wealth score), duration of illness, previous history of malaria episode in the last six months (self-reported), and the level of the facility where the patients visited significantly influenced the direct cost. For example, on average, for every additional kilometer of distance between the patients’ residence and the health facility, the direct cost increased by USD 0.27; and, for every additional day of illness the patient suffered, the direct cost increased by USD 0.41, but interestingly the severity variables were insignificant (Table 3).

**Table 3:** Quantile (median) regression of factors associated with variability of direct, indirect and total cost of malaria, Adami Tullu district south-central Ethiopia, 2015.

Cost of Malaria	Coef.	SE*	P-value*	[95% CI] *	
Direct Cost (n = 189, Pseudo R^2^ = 0.29)					
Wealth score	-0.222	0.063	< 0.001	-0.345	-0.098
Duration of illness (days)	0.413	0.171	0.010	0.077	0.748
Distance from home to the facility in km	0.271	0.064	< 0.001	0.146	0.396
Age of the patients	0.005	0.013	0.710	-0.020	0.030
Dummy for severe fever (ref = Mild)	-0.580	0.445	0.192	-1.452	0.291
Dummy for moderate fever (ref = Mild)	-0.884	0.653	0.176	-2.164	0.396
Treatment on the next day (ref = same day)	-0.235	0.376	0.533	-0.972	0.503
Treatment after two days and more (ref = same day)	0.378	0.341	0.268	-0.291	1.047
Received only prescription at PHCUs^¥^	-0.259	0.297	0.512	-0.842	0.324
Self-reported malaria episode last 6 month	-0.774	0.403	0.055	-1.563	0.015
Treated at health center (ref = treated at health post)	1.251	0.325	< 0.001	0.615	1.888
_cons	-0.048	0.781	0.951	-1.578	1.483
Indirect Cost (n = 141^£^, Pseudo R^2^ = 0.15)					
Wealth Score	-0.043	0.192	0.822	-0.419	0.333
Duration of illness	-0.653	0.513	0.203	-1.658	0.352
Distance from home to the facility in km	-0.096	0.116	0.408	-0.322	0.131
Age of the patients	0.092	0.065	0.157	-0.035	0.219
Dummy for severe fever (ref = Mild)	0.569	0.914	0.534	-1.222	2.360
Dummy for moderate fever (ref = Mild)	-0.860	1.822	0.637	-4.430	2.711
Treatment on the next day (ref = same day)	-0.200	1.392	0.886	-2.929	2.528
Treatment after two days and more (ref = same day)	-0.764	1.118	0.494	-2.954	1.426
Received only prescription at PHCUs	2.905	1.316	0.027	0.325	5.484
Had self-reported malaria episode last 6 month	-2.386	1.175	0.042	-4.689	-0.083
Treated at health center (ref = treated at health post)	-1.920	0.821	0.019	-3.529	-0.310
_cons	4.531	2.296	0.048	0.032	9.031

*Bootstrap standard error (SE), p-value and 95% confidence interval (CI) for the coefficient with 1000 replications.

^£^ We only estimate the indirect cost for age greater than 7;

^¥^ Primary health care units. ref = Reference category for dummy variables.

Likewise, the age of the patient, whether the patient received the drug directly from primary health care unit or sent out with only prescription (i.e availability of the antimalarial drug), history of malaria in the last six months, and the level of the facility visited significantly influenced the indirect cost. Among those treated at health centers, the direct cost was significantly higher, while the indirect cost was lower compared with those treated at health posts.

**Fig 1:**
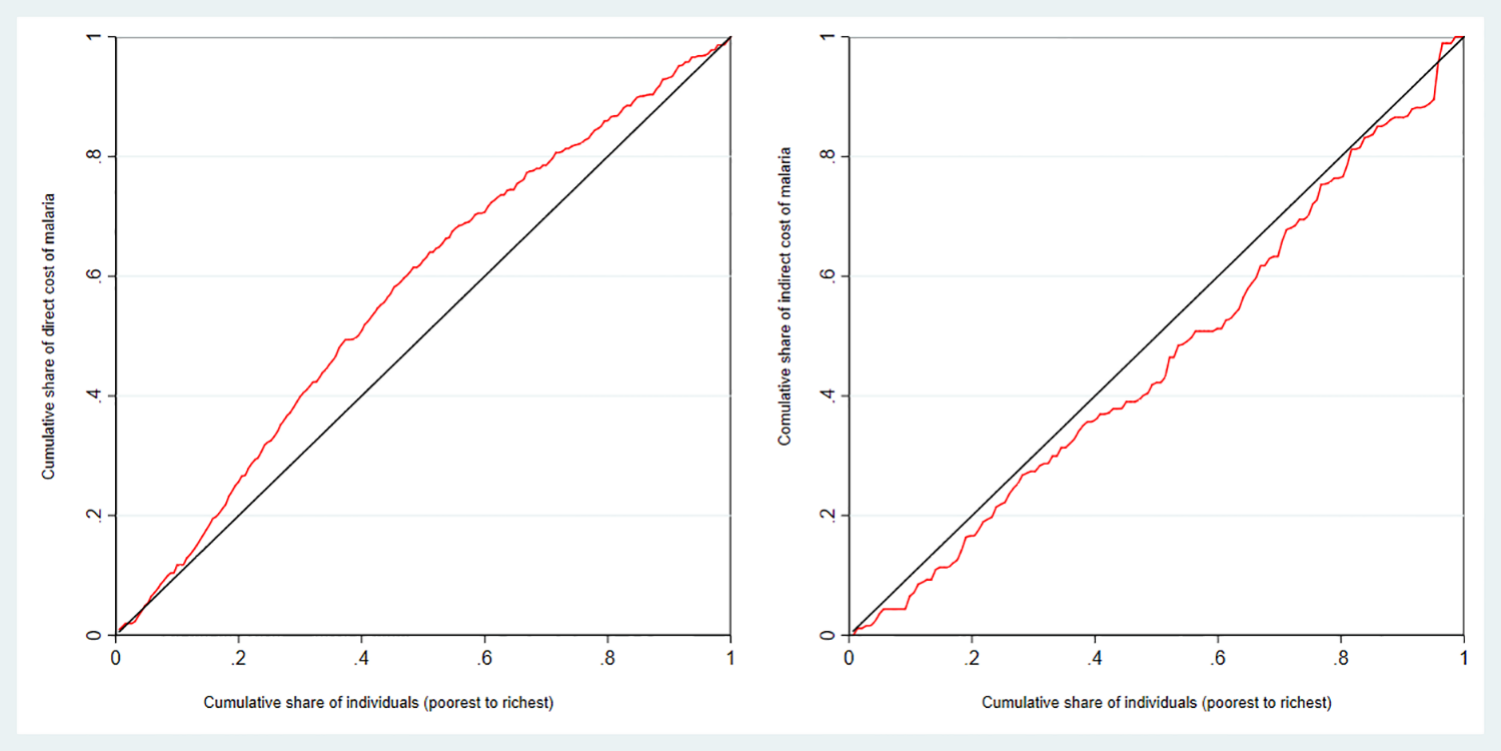
Concentrations curves for direct (A), and indirect (B) cost of malaria.

The mean and median cost distribution across wealth status is presented in Table 4. For the direct cost, the concentration curve (Fig 1A) and the concentration index of -0.155 (SE = 0.029, P < 0.001) indicates an inequity that patients from the poor households incur significantly higher cost (pro-rich distribution). However, the concentration index of 0.078 (SE = 0.059) and the concentration curve which was closely aligned with the diagonal line (Fig 1B) for the indirect cost distribution indicates that there was no noticeable difference in accordance with different socioeconomic status.

**Table 4:** Mean malaria costs and concentration indices across different socio economic status, Adami Tullu district in south-central Ethiopia, 2015.

Socioeconomic status	Direct cost		Indirect cost	
	Mean	Median	Mean	Median
Poorest	3.06	3.22	3.24	2.25
2^nd^ Poorest	3.01	3.22	3.67	3.63
Middle	2.37	2.37	3.10	1.88
2^nd^ Richest	1.82	1.66	4.67	4.00
Richest	1.67	1.05	3.99	1.45
Concentration Index (CI)	-0.155		0.078	
Standard error	0.029		0.059	
P-Value	< 0.001		0.185	
Kruskal Wallis test (P-value)	< 0.001		0.327	

Fifty-seven (30%) of the cases were diagnosed with *P*. *vivax*, while 133 (70%) were diagnosed with *P*. falciparum. Of those *P*. *vivax* cases, the history of malaria (self-reported) in the last six month were 25%, while it was only 18% among those *P*. *falciparum* cases. Table 5 illustrates the mean and median cost of malaria by species. *P*. *falciparum* is significantly costly for households, especially in terms of the indirect costs (Mann-Whitney test P <0.001).

**Table 5:** Analysis of the difference in median and mean cost of P. falciparum and P. vivax malaria, Adami Tullu district in south-central Ethiopia, 2015.

Cost of malaria Malaria species	Mean	Median	Mean	Median	Significance of the difference in median (Mann-Whitney test)	
	*P*. *falciparum*		*P*. *vivax*		Z	P-value
Direct Cost	2.16	2.20	2.92	3.07	-3.072	0.002
Indirect Cost	4.55	3.72	0.92	0.00	5.150	< 0.001
Total Cost	6.74	5.80	3.80	3.76	3.388	< 0.001

## Discussion

Transparent and data-driven evidence regarding the economic burden of malaria is more important than ever in this era of elimination and eradication [14] to inform prioritization of essential health service packages and policy decisions at national and regional levels. This study is the only one to provide empirical estimates regarding the economic burden of malaria to Ethiopian household in the last decade. In this study, we first estimated the economic burden of malaria in terms of direct and indirect cost to the rural household. Then, we identified predictors for variability in the cost.

We found that the median cost of malaria per episode to the household was USD 5.06. The direct cost accounted for nearly 40% and we found a significantly pro-rich inequality. In addition, socioeconomic status, distance between the patient’s residence and the health facility visited, incident of malaria in the last six months, level of the facility visited (health center versus health post) in the health system, and availability of the antimalarial drug in the health facility significantly influence either direct cost, indirect cost, or both.

The cost related to malaria episodes could be considered substantial to households in Ethiopia; where, according to World Bank report [35], more than a quarter of the total population is living in absolute poverty. The poverty situation is worse in the rural households [36]. The recurrent nature of malaria and a coincidence of malaria peak season with harvesting season accentuated the burden for the rural poor who are already dependent on subsistence farming and with limited coping options [8, 37]. The burden of malaria[38]

Comparing evidence of economic burden of malaria from different settings, time periods, patient groups, and epidemiological profiles is challenging [25]., Yet, several studies from various settings from African and Asian countries using different costing methods and patient-groups consistently found that the cost of malaria is substantial as we did [39–46]. To mention few, a population-based cost estimate from Sudan (Khartoum) among all age groups reports direct treatment expenditure of USD 6.3 and indirect cost per fully cured case of USD 3.2 [43]. A hospital-based estimate among children less than 3 year-old treated at outpatients from Asia, Papua New Guinea, reports ranging from USD 7.54 in one state (Madang) to USD 9.20 in another state (Maprik) [44].

Our cost estimates were slightly lower compared to most previous studies from Ethiopia [6, 21] or elsewhere [39, 40, 43, 44]. This might be due to several reasons. On one side, a recent policy change in introduction of Artemisinin-based Combination Therapy (ACT) and Rapid Diagnostic Testing (RDT) kits improved the malaria management in the country [47, 48]. This effective drug (ACT) and swift diagnostic method (RDT) likely have shortened the duration of the illness, and decreased both the direct and indirect costs [49]. On the other side, despite these drugs and the kit are quite expensive to the health system (provider), large-scale subsidization of these medicines in the public and private health facilities have decreased the patient’s costs compared with the previous sulfadoxine-pyrimethamine (*Fansidar*^®^) based regimen [50, 51]. Furthermore, malaria diagnosis and treatment have been directed more towards health post level by the health extension workers [7], which also reduce the total cost to some extent. According to a recent systematic review which includes several studies from sub-Saharan Africa, cost of malaria diagnosis and treatment is irregular and context dependent [40]. Changes in policy or technology (e.g. new malaria treatment guideline, new malaria diagnostic tool, new user-fee payment system, new malaria drug logistic system, etc.) is likely to change the cost of malaria at both patients and health systems level.

After all, we believe, a proper implementation of a day-to- day malaria management at all level of the health system and every health facility is more crucial to provide affordable and swift service to the suffering patients. For instance, the current study show indirect cost was mainly influenced by availability of the antimalarial drug in the health facilities. On average, those patients examined and diagnosed with malaria but sent back home with only prescription paper—without a drug—had incurred about USD 2.9 higher indirect cost compared with patient received the drug directly from the public primary health care facilities. Most likely, either these patients had spent long time searching for anti-malaria drug from a private drug store/pharmacy or they stayed at home without any access to treatment. In both cases, these patients were prone to delayed treatment, longer duration of illness, and expensive and counterfactual drugs [52]. In fact, Federal Ministry of Health of Ethiopia commissioned evaluation indicates that stock-out of essential drugs (i.e. including malaria drugs) from public health care facilities is very common and the average stock-out duration is about 100 days [53]. Although this study is somehow older, there is less evidence which proves the improvement of pharmaceutical supply system in Ethiopia within this period [52].

Cost information disaggregated by level of hierarchy in the health system is quite important and provides an opportunity for in-depth analysis of the policy options. In the Wilcoxon rank-sum test, we found that the difference in total cost between health post and health center was not significantly different from zero (P = 0.291). Similarly, the median indirect cost at health center was not significantly different from its counterpart at health post (P = 0.29). However, the direct cost at health post was significantly lower compared with direct cost at health center (P < 0.001) health post In line with the national health sector transformation plan, malaria diagnosis and treatment services at health post shall be free of user-fee charges [51]. However, it is not necessarily meant that the direct costs at health post were negligible; given that the non-medical cost attached to transportation, food, and other items were palpable (Table 2).

Those who had previous malarial illness in the last six month (i.e self-reported malaria) incurred significantly lower cost, mainly in the indirect cost. On average, those who had malaria in the last six month incurred USD 2.4 less indirect cost compared with those had not. This could be due to different reasons: self-medication with ‘leftover’ drugs [21, 54]; improved resilience, better coping mechanisms, and better informed from the experience of the recent illness [55]. To some extent, it was due to most of the recurrent cases of malaria being *P*. *vivax*, which is less severe and less costly as we found in this study (Table 5). This needs again further research to look the interaction between disease recurrences, health care seeking behavior, resilience, and productivity.

The bivariate analysis, the multiple quantile regression, the concentration index, and the concentration curve consistently indicate that the household’s socioeconomic status significantly influenced the direct cost, while the influence was consistently not statistically significant for the indirect cost. Poor shouldering the highest financial burden against their limited ability to pay is a striking finding. Out-of-pocket payments for malaria treatment can impoverish some households who are already on ‘border-line’ when it becomes recurrent and catastrophic in size, especially in a health system running without any mechanism for financial risk protection [9, 17, 38, 56]. When the share of out-of-pocket payments is greater than 10% of the total expenditure/income, the risk of the health expenditure to be catastrophic in size is very high [38].

On the other side, in the quantile regression, severity of the illness was not a significant predictor of neither direct costs nor the indirect cost. This might be mainly because of two methodological challenges: First, the severity classification method applied was reliant on self-reported fever, which is more prone to misclassification, and recall bias, might underestimate the true association between costs and severity to some extent. Second, in this study, we only include uncomplicated malaria cases and large majority (90%) of them had only mild to moderate level of fever. The area is also one of malaria endemic area; and, sever and complicated cases are less likely to occur because of resilience of the community to malaria developed along period. Hence, it is more challenging to capture adequate variability in terms of severity in the first instance. Otherwise, the cost of sever and complicated malaria is hugely larger than mild and uncomplicated cases. Hence, we suggests an in-depth estimation of the cost of sever and complicated malaria; such studies would have a paramount importance.

Similarly, the indirect cost was not consistent and not significantly different across wealth quantiles. Indirect cost, in this study, was entirely an estimate of working day lost which is homogenous across wealth quantile and could be affected by several interconnected of factors. Working day lost by the patents should be influence by the extent of the illness in terms of severity and duration in addition to the response/ reaction of the patients and the family to the illness, for example, some patients might stay at home while some other patients stay at work irrespective of the severity of the illness. To some extent, treatment and diagnostic service provided, for instance, some patients might got the service in the nearby facility while some might need to travel far and spend additional days off work seeking the service, might influence the indirect cost.

This study provides empirical evidence based on patient level data. However, selection of participants was done at health facilities with careful considerations to included households from divert socioeconomic and demographic background to make results representative for rural households in the most part of Oromia region, if not Ethiopia. Despite all efforts, indisputably, cases identified from health facilities are usually different from what could have been if we used a community-based household survey. To some extent, this could affect our cost estimates although it is difficult to speculate the direction of the influence. Furthermore, to avoid over/underestimation because of the seasonality nature of malaria, we collected data for one full year. We also applied a multiple quantile regression method to produce standard errors that are more robust to outliers than ordinary least squares regression.

This study has some limitations that require results to be interpreted with care. Initially we assumed to include 260 participants, but the sample size was revised based on the preliminary analysis of the first 100 samples. Given that, we include adequate number of participant to estimate the cost with reasonable margins of error and standard deviation. However, our sample size might not be sufficient to testing hypotheses or to identifying some of the associated factors (e.g severity and immediate treatment seeking behavior). For instance, although this study did not find significant association between direct costs and malaria history in the last six months at 95% CI, some of the regression coefficients are non-negligible in size and could have become significant with large sample size (Table 3).

The other limitation of our study is that the assumption in wage rate estimation we employed in this study for teenagers (half of adults’ wage) and for children (quarter of adults’ wage) should have been cross-validated using local data from the study area or from other comparable districts. The involvement of teens and children in household chores and the responsibilities they take might be somehow different from place to place. In addition, the assumption we employed to convert the workdays lost into monetary value did not account for individual-level variations in actual or potential earning within the same age-group. The same value of labor (i.e the average wage rate for agricultural worker) was considered for patients within the same age group. The accuracy of our estimates may therefore depend on local variability of factors such as primary school coverage. Measuring indirect cost is a challenging exercise, especially in situations where labor markets are poorly defined, self-employed farming is the primary occupation of most households (90%), and seasonal variability of wage rate is high.

Finally, in this study we only considered costs associated with the current episode of malaria to the household, and we did not take into account long-term cost implications from complications, such as anemia, neurological sequel, cognitive loss, loss in school performance and future employability. A compressive study from the societal perspective could give a more complete result [25].

## Conclusion

In conclusion, the economic costs of malaria to households in rural Ethiopia represent a potentially high economic burden, mainly to the poor. An implication is that reducing malaria burden could contribute also to poverty reduction as well. Both provider and demand side factors influence the amount of direct and indirect cost. The national malaria program needs to recognize this economic burden and identify mechanisms for ensuring that the poor have uninterrupted easy access to malaria treatment services largely either subsidized or free of charge. The results of this costing study can be used as input to a full economic evaluation of the prevention of malaria in Ethiopia.

## Supporting information

S1 File Cost of malaria. (XLS)
